# Sink property of metallic glass free surfaces

**DOI:** 10.1038/srep08877

**Published:** 2015-03-16

**Authors:** Lin Shao, Engang Fu, Lloyd Price, Di Chen, Tianyi Chen, Yongqiang Wang, Guoqiang Xie, Don A. Lucca

**Affiliations:** 1 Department of Nuclear Engineering, Texas A&M University, College Station, TX 77843, USA; 2 Department of Materials Science & Engineering, Texas A&M University, College Station, TX 77843, USA; 3 School of Physics, Peking University, Beijing 100871, P.R. China; 4 Los Alamos National Laboratory, Los Alamos, New Mexico 87545, USA; 5 Institute for Materials Research, Tohoku University, Sendai 980-8577, Japan; 6 School of Mechanical and Aerospace Engineering, Oklahoma State University, Stillwater, Oklahoma 74078, USA

## Abstract

When heated to a temperature close to glass transition temperature, metallic glasses (MGs) begin to crystallize. Under deformation or particle irradiation, crystallization occurs at even lower temperatures. Hence, phase instability represents an application limit for MGs. Here, we report that MG membranes of a few nanometers thickness exhibit properties different from their bulk MG counterparts. The study uses *in situ* transmission electron microscopy with concurrent heavy ion irradiation and annealing to observe crystallization behaviors of MGs. For relatively thick membranes, ion irradiations introduce excessive free volumes and thus induce nanocrystal formation at a temperature linearly decreasing with increasing ion fluences. For ultra-thin membranes, however, the critical temperature to initiate crystallization is about 100 K higher than the bulk glass transition temperature. Molecular dynamics simulations indicate that this effect is due to the sink property of the surfaces which can effectively remove excessive free volumes. These findings suggest that nanostructured MGs having a higher surface to volume ratio are expected to have higher crystallization resistance, which could pave new paths for materials applications in harsh environments requiring higher stabilities.

Metallic glasses (MGs) exhibit unique mechanical and chemical properties due to the absence of long range order and grain boundaries^1^,^2^,^3^. The MG phase is metastable and a transition from complete amorphous to partial crystallization occurs when highly correlated atomic movements are allowed. Nucleation and growth of nanocrystals have been observed under heating^4^, ion/electron irradiation^5^,^6^,^7^, bending^8^, indentation^9^, and pressure^10^. One key to induce athermal nanocrystallization is to introduce excessive free volume (FV) whereas positive FV means more open space. FV can be treated as “flowing defects” in MGs, based on the fact that FV contributes to macroscopic atom transport in the free volume model^11^,^12^,^13^, and that FV at sufficiently high levels can induce a localized amorphous-to-crystalline transition. The latter resembles crystalline solids in which point defects above critical densities can induce a crystalline-to-amorphous transition^14^.

In crystalline solids a large supersaturation of point defects cannot develop in the presence of high defect sink densities. Various nanostructured crystalline metals having large surface/interface areas were found to have enhanced defect-self-repairing capabilities^15^,^16^,^17^,^18^,^19^. Can free surfaces of MGs have similar defect sink properties to remove FV? If true, novel structural engineered MGs might open new doors for obtaining higher stability and higher crystallization resistance. MGs in general have relatively low T_g_ (glass transition tempereature) which greatly limits their applications. When heated up to a temperature close to T_g_, significant crystal nucleation starts^20^. If heated with concurrent ion irradiation, the temperature for nanocrystallization is even lower (to be shown in the present study). If crystallization resistance can be systematically increased, MG applications can be further extended into harsh environments. Examples include MG coating of underground oil pipes or spent nuclear containers in deep geological disposal, or even fuel cladding in reactors.

We used in situ transmission electron microscopy (TEM) with concurrent heavy ion irradiation and annealing to study the microstructural changes of Zr_50_Cu_35_Al_7_Pd_5_Nb_3_ MG membranes prepared by electropolishing. This particular MG was selected due to its high glass-forming ability and unusual plasticity^21^. The experiments were conducted at the IVEM-Tandem National User Facility at Argonne National Laboratory. A 1 MeV Kr beam was introduced into the TEM chamber, bombarding the MG specimens at 30° off the electron beam direction.

Figure 1A shows the TEM micrographs and selected area diffraction (SAD) patterns obtained from the MG with the irradiation temperature kept at 693 K, which is lower than the T_g_ (710 K) measured from differential scanning calorimetry (see supplementary Fig. S1). The Kr ion fluence was incrementally increased, and the beam was stopped for a short time period to allow for structural relaxation and TEM imaging. When the Kr ion fluence reaches 4.3 × 10^13^ Kr/cm^2^ and above, nanocrystals appear in the TEM micrographs and white spots appear in the SAD patterns. From a systematic study, Fig. 1B summarizes the temperature vs. Kr ion fluence required to initiate nanocrystallization. The solid line is a linear fit of the data and is used as the boundary to separate the irradiation stable and unstable regions. Clearly, increasing Kr ion fluence reduces the temperature required for nanocrystallization.

The most interesting finding is that MG membranes of different thickness exhibit different crystallization resistance. It is typical to find that MG pores created from electropolishing have ultra-thin edges, which appear with relatively brighter contrast in a bright field TEM micrograph. The thickness of this ultra-thin edge is <15 nm, estimated from electron energy loss spectroscopy. Next to this rim is a relatively thicker region which appears with darker contrast. This thicker region is still electron transparent and its thickness is estimated to be around 100 to 200 nm. As shown in Fig. 2A, at 733 K and 7.5 × 10^13^ Kr/cm^2^ the thick region began to develop nanocrystals, but the ultra-thin region was still completely amorphous. The micrograph observations agree with the SAD results, as shown in Fig. 2B, in which the thick region shows white spots while the ultra-thin region still maintains amorphous features. When the temperature is further increased to 813 K and the fluence to 1.0 × 10^14^ Kr/cm^2^, the ultra-thin region begins to develop nanocrystals, as evidenced by the SAD patterns. At higher temperature and higher fluences, nanocrystals in both regions are formed. The study shows that the ultra-thin MG region forms nanocrystals at a temperature which is 120 K higher than that of the irradiated thick MG, and 103 K higher than the T_g_ of unirradiated MG.

We can use the nucleation rate within the supercooled liquid region as invariance to establish a linkage between various experiments, thus deriving an equation to describe the nanocrystallization temperature shift as a function of ion fluence. Under a steady-state nucleation condition, the nucleation rate, *r*, is given by^3^

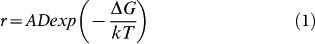
where A is a constant, D is effective diffusivity, k is the Boltzmann constant, T is temperature, and Δ*G* is the activation energy barrier for forming stable nuclei. The activation energy barrier Δ*G* in Eq. [1] can be expressed as 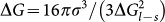 3, where *σ* is the interfacial energy and Δ*G_l-s_* is the free energy difference between a liquid state and a crystalline state. The diffusivity D, which originates from the free volume model^23^,^24^, can be calculated by 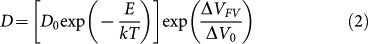
where *D*_0_ is a constant, *E* is mean migration energy, Δ*V_FV_* is excess free volume, Δ*V*_0_ is some activation volume. Assuming a critical r value is required for nanocrystallization, Eqs. [1] and [2] lead to the following approximation near the *T_g_* region: 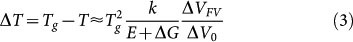
where Δ*T* is proportional to Δ*V_FV_* and therefore proportional to the Kr ion fluence. This linear dependence is observed in Fig. 1B. At higher ion fluences if positive FV and negative FV are allowed to dynamically recombine in a manner similar to annihilation of interstitials and vacancies in crystalline solids, excess free volume may saturate and the dependence of Δ*T* on ion fluence may deviate from a linear relationship, which is to be verified in a future study.

The above discussion assumes that the irradiation effect on Δ*G* is less prominent. The mechanism of Δ*G* change is complicated since it is determined by the enthalpy, entropy, and specific heat capacity difference between crystalline and amorphous states^25^. If ion irradiation induces phase segregation, entropy will drop due to the reduced number of microscopic states^26^, thus increasing Δ*G_l–s_*. Previous studies on different MGs have observed the agglomeration of nanocrystals of a new phase and subsequent formation of stable nuclei from the agglomeration^27^, but such phase separation was not observed in the present study and will not be a significant driving force for nanocrystallization. Furthermore, introducing excess free volume will decrease the specific heat capacity and thus increase Δ*G_l–s_* although the effect is small.

We conclude that the MG nanocrystal formation observed in the present study is caused by the excessive FV introduced by ion irradiation, instead of localized crystallization along the ion track. Under heavy ion irradiation such as with Kr ions, the MG membrane can have both scattered displacements and damage cascades forming along the ion track. A damage cascade has a relatively empty core surrounded by displaced atoms. After a few picoseconds, the kinetic energy of displaced atoms is converted to local heat, leading to thermal spike formation, with temperature in the core high enough to melt MGs. However, the subsequent thermal dissipation is very fast, with a cooling rate over 10 orders of magnitude higher than the critical cooling rate for MG formation, as reported in our previous study^22^. Therefore, the molten region reforms MG. Further evidence that the ion track does not lead to direct crystallization is that the number of nanocrystals formed does not increase with increasing Kr ion fluence. In one set of experiments performed with simultaneous Kr ion irradiation and TEM imaging, the number of nanocrystals formed under continuous irradiation was found to saturate. Furthermore, growth of existing nanocrystals into bigger sizes and diminishing of existing nanocrystals were observed (see supplementary Fig. S2). This observation suggests that the nanocrystals require a critical size to reach stability and continuous growth.

The study involved prolonged electron beam analysis. Although previous studies have shown that electron beams can induce nanocrystals, we don't believe this is the case here due to low beam current and low electron energy used. First, electron beam heating was estimated to be about 2 K (see supplementary), which is ignorable. Second, the TEM electron flux in the present study was two orders of magnitude lower than that used in previous studies which intentionally used a strongly focused electron beam to induce crystallization^5^. To further evaluate the portion of nanocrystallization caused by the electron beam, we measured the nanocrystallization temperature under concurrent heating and TEM analysis, but without Kr ion irradiation. This temperature was determined to be 703 K, which is 7 K below the T_g_ (see supplementary Fig. S3). The data was plotted in Fig. 1B corresponding to zero Kr ion fluence. The small temperature difference suggests that the electron beam plays a small, if not ignorable role.

The present study shows that MG is similar to a nanostructured crystalline solid in which free surfaces can serve as defect (FV) sinks. MG free surfaces are expected to be able to remove both positive FV and negative FV, thus leading to higher crystallization resistance. Additional evidence of this FV sink property of MG surfaces is provided through our MD simulations. Due to the complexity of the interatomic potential pairs required to describe a true multi-element MG, the present modeling used a pure amorphous Ni film for simplicity. The Ni amorphous structure is created by first heating a crystalline Ni film up to 4000 K and then quenching it down to 1 K instantly to freeze its amorphous nature. The film is then slowly heated from 1 K to 600 K for structural relaxation. At time t = 0, the relaxed film is then introduced with FV (Δ*V_FV_* > 0 by randomly removing 20% of the atoms within the whole film. For atoms around a removed one, their larger atomic separation distances lead to higher potential energies. Therefore, the FV contained region can be visually mapped by plotting the atoms having potential energies above a certain value (−3.8 eV), as shown in Fig. 3A. With increasing annealing time at 600 K, FV is gradually removed, starting first from the film surfaces. At time t = 10 ps, the majority of FV is removed. The surface atoms always have higher potential energy. FV removal also leads to shrinkage of the film thickness. Figure 3B plots FV distributions, obtained by calculation of Δ*V*/*V*, where *V* is the mean volume occupied by one Ni atom in a relaxed amorphous film and Δ*V* is the amount of volume increased due to FV introduction. The original box-like FV distribution evolves into a Gaussian like peak at longer times, and eventually the peak diminishes. Such changes can be approximated by diffusion-mediated FV spreading under the boundary condition that excessive FV is fixed to be zero at the two surfaces.

The likelihood of crystallization can be quantitatively represented by time duration when FV is kept above a critical value. We therefore introduce *τ*_1/2_ as the time required for the film center to reduce down to the half of its original value at t = 0. Figure 3C plots the *τ*_1/2_ as a function of the film thickness. Considerable FV removal in the center occurs when the FV diffusion length becomes comparable to half of the film thickness *d*, as approximated by 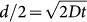. This approximation agrees with Fig. 3C in which the data trend suggests *τ*_1/2_ ∝ *d*^2^. Longer *τ*_1/2_ in a thicker film gives a higher possibility to develop correlated atomic rearrangements, and the opposite occurs for an ultra-thin film.

The study suggests a path to systematically enhance resistance to crystallization for MGs by using surfaces or interfaces as sinks to remove FV, or as annihilation centers to combine positive and negative FV. Since FV fluctuations can be introduced by thermal annealing or electron/ion irradiation, the proposed structural engineered MGs can have longer lifetime under harsh environments involving higher temperature or particle irradiation. The FV sinks are not necessarily only free surfaces. Interfaces of different MGs can also function as sinks. Interface reactions between different MG systems may reduce MG stability but issues may be alleviated by introducing interfaces of immiscible systems. It is also attractive to introduce multilayered structures with alternatively deposited MG and other advanced highly stable materials such as newly identified ceramics which can withstand high temperature over 1,773 K^28^.

## Methods

### Sample synthesis and TEM specimen preparation

During sample synthesis, a ribbon Zr_50_Cu_35_Al_7_Pd_5_Nb_3_ alloy sample, 1.5 mm wide and 20 *μ*m thick, was fabricated by melting mixtures of pure metal powders and rapid solidification of the melt on a copper roller in an argon atmosphere. A jet electro polisher set up was used to prepare TEM specimens. The polisher reservoir was first filled with a mixture of 75% methanol 25% nitric acid to a level that comfortably covered the suction intake of the pump. This mixture was cooled using liquid nitrogen until the temperature was measured to be below 258 K. The sample was then cut to length, ≈5 mm, and mounted into the holder. With the setup complete, the sample was repeatedly polished in intervals from 2–10 seconds, inspecting between each polish under an optical microscope, until a hole was seen to develop in the metallic glass sample. This indicated that the sample was electron transparent, and ready for use in the TEM.

### Ion irradiation and in situ TEM characterization

The in situ TEM characterization with concurrent ion irradiation was performed by using the IVEM Tandem National User Facility at Argonne National Laboratory (ANL). The angle between the incident electron beam and the ion beam was about 30 degrees. The IVEM Tandem is an intermediate voltage microscope interfaced with a 2 MV ion accelerator. A Kr beam of 1 MeV energy was rastered over the MG specimen to guarantee uniformity. Prior to ion irradiation, specimens were slowly preheated to the designed temperature. All TEM micrographs and diffraction patterns were collected when the Kr ion beam was off. A movie showing dynamic nanocrystal changes was taken when the Kr ion beam was on. Position shifting occurred during heating and ion irradiation, therefore position adjustments were always needed and it was difficult to perform characterization on the same spot.

### Molecular dynamics simulations

MD simulations were performed by using the LAMMPS code^29^. An empirical EAM potential was used to describe Ni interatomic interactions^30^. Amorphous structures were obtained by heating up to 4000 K for 0.5 ns, and cooling down to 1 K instantly. The structures were then slowly heated from 1 K to 600 K to allow for structural relaxation. To create free volume, 20% of the atoms were randomly selected and removed from the cell. The subsequent structural changes were modeled at a constant temperature of 600 K, under a NVT ensemble. MG membranes of different thickness, 5.3, 10.6, 15.8 and 21.1 nm, were modeled. The thicknesses correspond to 15, 30, 45, 60 unit cell lengths in the starting Ni super cells.

## Supplementary Material

Supplementary Information Supplementary Information

## Figures and Tables

**Figure 1:**
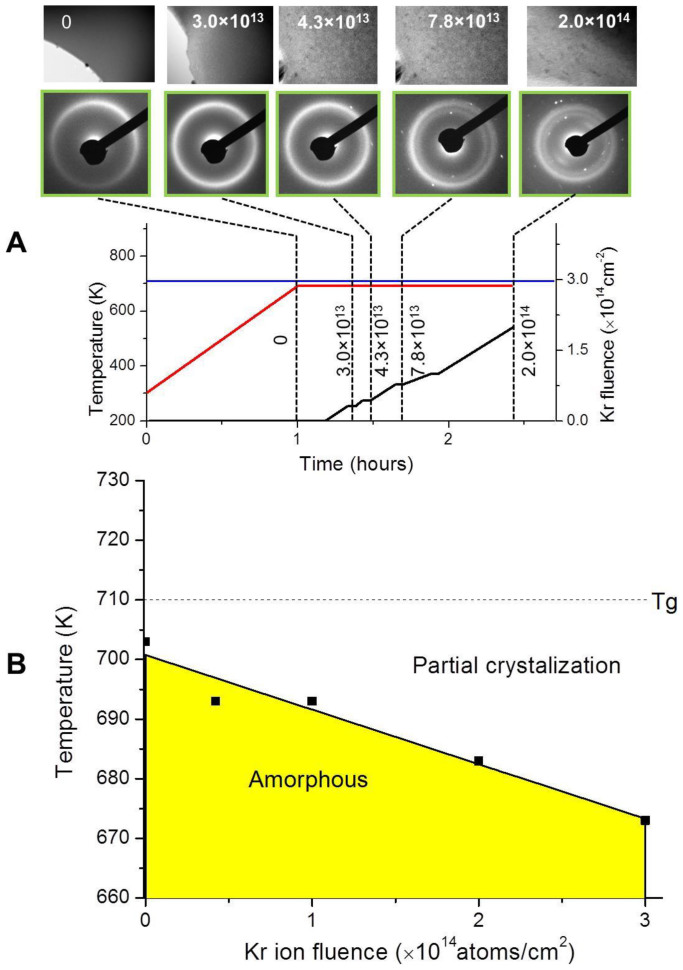
Bright field TEM micrographs and corresponding SAD patterns obtained from one irradiation experiment and the summary of the stability boundary for various irradiation conditions. (A) The selected SAD patterns are collected after different fluences of Kr ion irradiation, as guided by the dash lines. The ion irradiation starts when the annealing temperature reaches 693 K. The red curve shows the time dependent temperature changes and the black line shows the time dependent Kr ion fluences. The blue line shows the glass transition temperature T_g_ measured by using DSC in bulk MG. (B) The yellow region shows the annealing temperature and Kr ion fluence under which MG membranes still remain complete amorphous structures without nanocrystallization. The dashed line shows T_g_. The solid line defining the yellow region is obtained from a linear fit of the solid symbols, which are experimentally determined.

**Figure 2:**
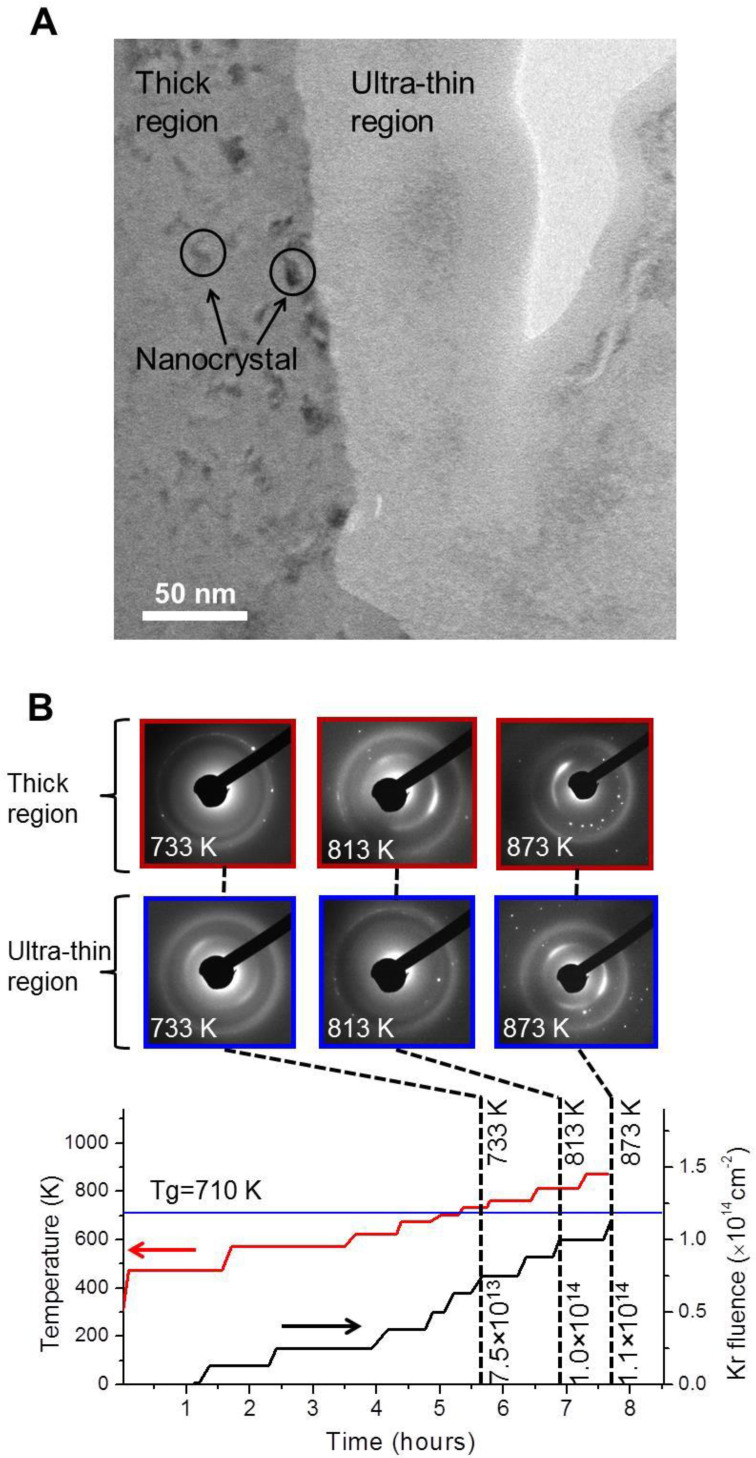
TEM micrograph and SAD patterns obtained from Kr ion irradiated MG membranes. (A) TEM micrograph of the MG membrane irradiated up to 7.5 × 10^13^ Kr/cm^2^ and heated up to 733 K. The dark region corresponds to a relatively thicker membrane and the middle bright region corresponds to a relatively thinner membrane. Circles point out typical nanocrystals observed in the thick region, while the ultra-thin region has no nanocrystals observed. (B) The selected SAD patterns collected at different stages of heating and ion irradiation, as guided by the dash lines. The red curve shows temperature changes and the black curve shows Kr ion fluence changes. The first row of SAD patterns were collected from the thick region, and the second row of SAD patterns were collected from the ultra-thin region, as labeled in (A). For the thick region, white spots appear in all SAD patterns from the thick region; for the ultra-thin region, white spots appear only when the temperature reaches 813 K and above.

**Figure 3:**
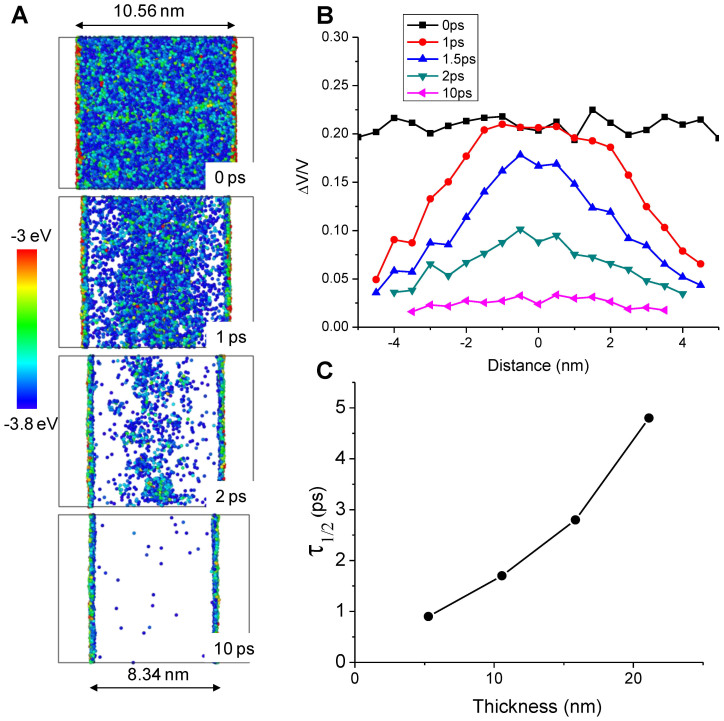
Molecular dynamics simulations of an amorphous Ni film. (A) The plot of atoms having potential energy above −3.8 eV, as a function of annealing time at 600 K. At time t = 0, 20% atoms are randomly removed to introduce FV. Arrows refer to the film thickness. (B) The plot of Δ*V*/*V* as a function of annealing time, where *V* is the mean volume occupied by one atom. (C) The plot of required time to reduce Δ*V*/*V* by half in the film center as a function of film thickness.
