# Supplementary material for: Sink property of metallic glass free surfaces
Source: Sci Rep. 2015 Mar 16;5:8877. doi: 10.1038/srep08877 (PMC5390901; doi:10.1038/srep08877)
Supplement: Supplementary Information [file srep08877-s1.pdf]

Supplementary information for

Sink property of metallic glass free surfaces

Lin Shao[1,2], Engang Fu[3], Lloyd Price[1], Di Chen[1], Tianyi Chen[1], Yongqiang Wang[3], Guoqiang Xie[4], Don A. Lucca[5]

[1] Department of Nuclear Engineering, Texas A&M University, College Station, TX 77843, USA

[2] Department of Materials Science & Engineering, Texas A&M University, College Station, TX 77843, USA

[3] Los Alamos National Laboratory, Los Alamos, New Mexico 87545, USA

[4]Institute for Materials Research, Tohoku University, Sendai 980-8577, Japan

[5]School of Mechanical and Aerospace Engineering, Oklahoma State University, Stillwater, Oklahoma 74078, USA

DSC measurement of as-cast metallic glass

Figure **S1** shows a DSC curve for as-cast $Zr_{50}Cu_{35}Al_7Pd_5Nb_3$ metallic glass, measured at a heating rate of 0.67 K/s. The glass transition temperature, the temperature where a transition from glassy to supercooled liquid occurs, was determined to be 710 K. The DSC curve also shows that the onset of the first-step crystallization is at 770 K. The temperature difference between the glass transition temperature and the crystallization temperature, the so-called width of the supercooled liquid region, is a gauge to evaluate the thermal stability against crystallization [31]. The width of 60 K suggests that the selected MG is intermediate for its crystallization resistance under electron or ion beam irradiation, which is one of the primary reasons that this particular type of MG was selected for the present study.

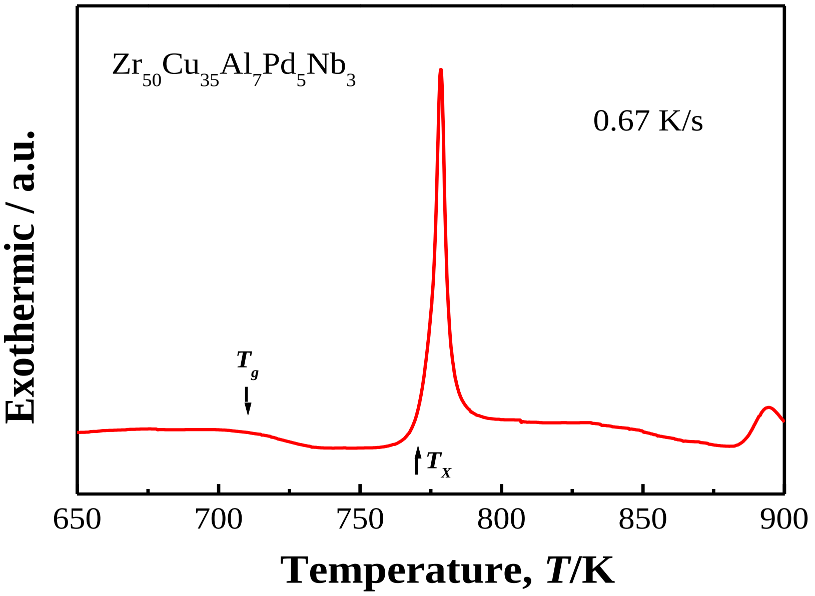

Fig. S1. DSC trace of the as-cast $Zr_{50}Cu_{35}Al_7Pd_5Nb_3$. The DSC measurement was performed on an as-cast MG ribbon sample.

Nanocrystal interactions under heating and concurrent Kr ion irradiation

We recorded the dynamic response of the nanocrystals under Kr ion irradiation. Figure **S2** shows snapshots of dark field TEM micrographs with increasing Kr ion irradiation time. The time t=0 s corresponds the starting point when the Kr ion fluence reached 4.04×10^{14} cm^{-2} and heating temperature reached 683 K. The Kr ion bombardment continued during TEM imaging. At t=0 s, nanocrystals were formed and appear as white spots. The blue and red arrows mark two nanocrystals close to each other. At t=49 s, one nanocrystal (red) became smaller while the other (blue) became larger. At t=78 s, the shrinking nanocrystal disappeared and the growing nanocrystal was bigger. At t=103 s, the growing nanocrystal was still present with seemingly larger size.

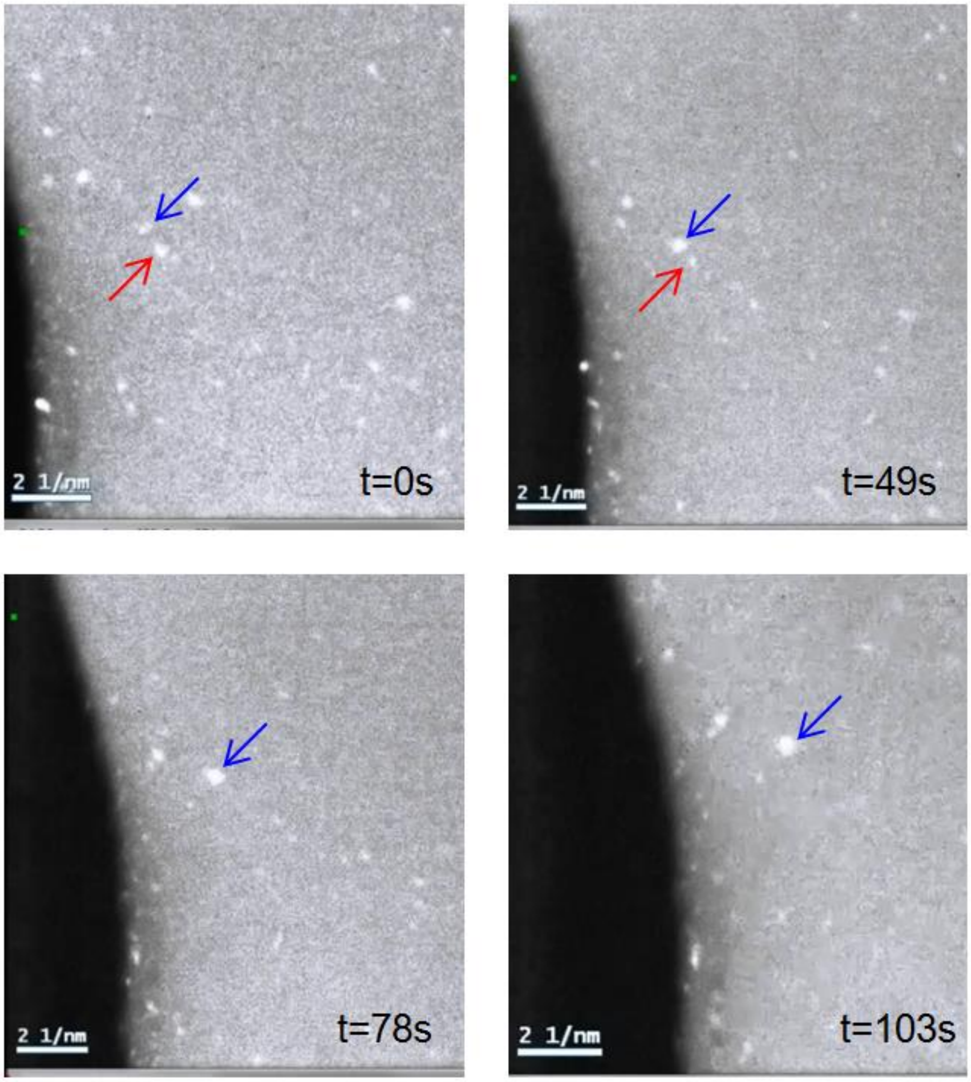

Fig. S2. TEM dark field micrographs of MG heated at 683 K with concurrent Kr ion irradiation. The blue and red arrows are used to mark two traced nanocrystals. The time t=0 s corresponds to a Kr ion fluence of $4.04\times10^{14}cm^{-2}$. The time t=103 s corresponds to a Kr ion fluence of $4.16\times10^{14}cm^{-2}$. The temperature is kept at about 410 K.

Nanocrystallization under the TEM electron beam without Kr ion irradiation

Figure **S3** shows the selected area diffraction (SAD) patterns collected when the MG is heated incrementally from room temperature up to 714 K. Here there was no Kr ion irradiation and the study was used to evaluate the thermal stability under a combined effect of annealing and irradiation from the TEM

electron beam. At a temperature of 623 K, no crystallization was observed and the SAD pattern featured maze-like halo rings. At a temperature of 703 K, the SAD pattern began to show white diffraction dots. Previous studies have shown that a high intensity focused electron beam is able to induce crystallization under room temperature, i.e., the electron flux is about 7×10^{23} $m^{-2}s^{-1}$ [5]. Different from the previous studies, the electron flux used in the present study was not focused and was kept at 5×10^{21} $m^{-2}s^{-1}$, which was about two orders of magnitude lower. Therefore we did not expect that the onset temperature to induce nanocrystals would be significantly reduced. On the other hand, the temperature of 703 K is 7 K below the glass transition temperature, which suggests that the electron beam played a minor role in promoting nanocrystals. For this reason, the temperature of 703 K was used as the base line to discuss the nanocrystal formation temperature shift when the Kr ion beam was used.

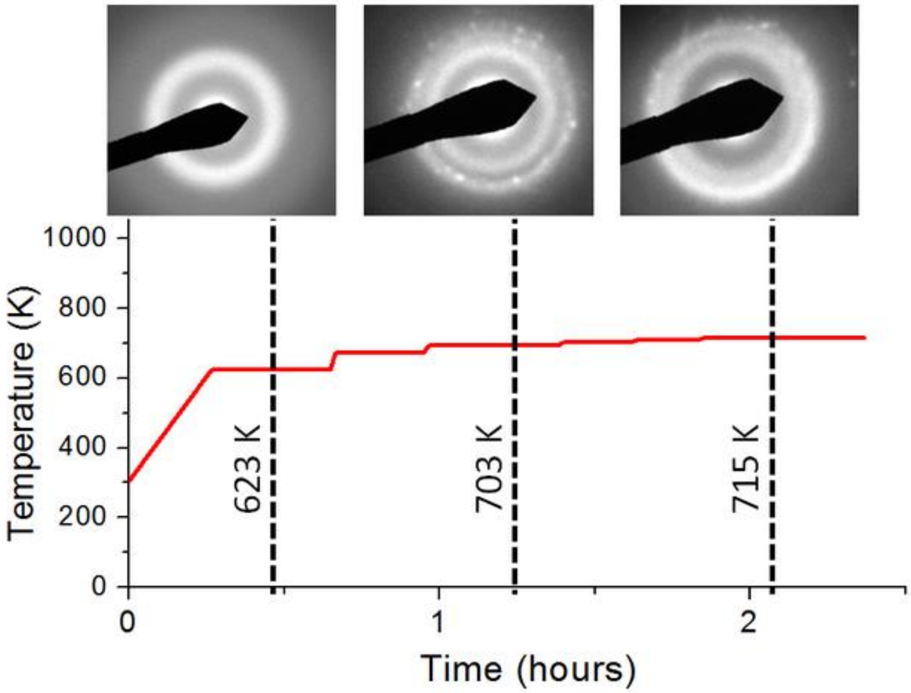

Fig. S3. The temperature changes as a function of time for the MG exposed under the TEM electron beam. Three selected SAD patterns are used as the insets to show that the onset of nanocrystallization occurs at 703 K.

Beam heating caused by the electron beam

The observed nanocrystallization is not caused by electron beam heating. The temperature rise under an electron beam can be estimated by the following equation [32].

$$\Delta T = \frac{I}{\pi k e}\left|\frac{dE}{dx}\right|\ln\frac{R}{r_0} \qquad [S1]$$

where k is thermal conductivity ($k \approx 10W/mK$), R is the TEM specimen radius $(R \approx 2mm)$, r_0 is electron beam radius $(r_0 \approx 200nm)$, I is electron beam current $(I \approx 10nA)$, and *dE/dx* is the electron stopping power.

The *dE/dx* of electrons can be calculated by [33]

$$\left|\frac{dE}{dx}(keV/cm)\right| = 7.85\times10^4\frac{Z\rho}{AE}\log\left(\frac{1.166\times200}{J}\right)$$
$$J(keV) = (0.76Z + 58.5Z^{-0.19})\times10^{-3} \qquad [S2]$$

where Z is the averaged atomic number, ρ is the density ($\rho \approx 7g/cm^3$), A is the atomic weight (g/mole), and E is beam energy (E=200 keV). Eq. [S2] gives $|dE/dx|$=4514 keV/cm.

Combining Eqs. [S1] and [S2] gives a temperature rise of 2 K from the electron beam. Therefore, it can be concluded that electron beam heating is negligible.

Amorphous status of MD simulated Ni film before and after FV removal

Figure S4 compares radial pair distribution functions (RDF) of Ni. Figure S4(a) corresponds to perfect crystalline Ni, which is featured with sharp peaks due to well defined lattice distances. Figure S4(b) corresponds to amorphous Ni after structural relaxation but prior to introducing FV. The film thickness is 10.56 nm. The shortest pair distance is larger than that of crystalline Ni, and all peaks are broader, as expected from an amorphous structure. Figure S4(c) corresponds to amorphous Ni after FV is removed via free surfaces, which is almost visually indistinguishable from (b). Due to the surface defect sink properties, the quick annihilation of FV defects helps to sustain the amorphous structure, which is evidenced by the lack of any sharp peaks as observed in Fig. S4(a).

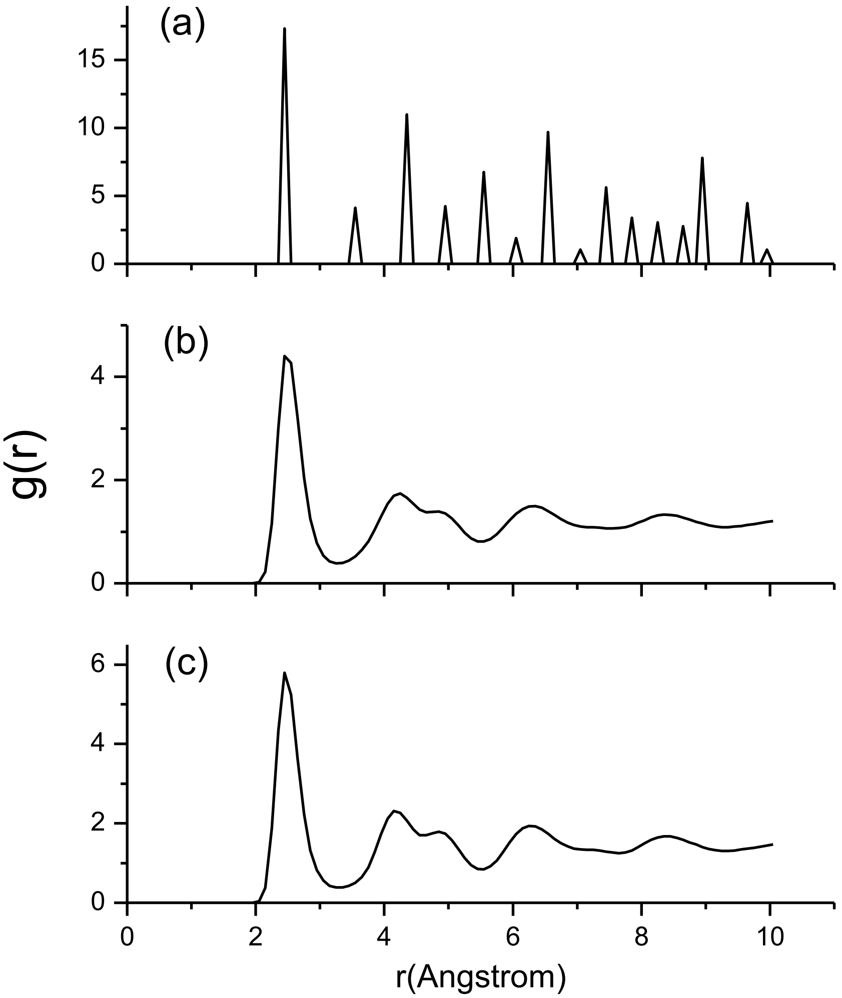

Fig. S4. A comparison of radial pair distribution functions (RDF). (a) A crystalline Ni film of 10.56 nm thickness. **(b)** A amorphous Ni film prior to random removal of Ni atoms to introduce FV defects. **(c)** The amorphous Ni film after FV defect removal by the surfaces, at time t=10 ps and annealing temperature of 600 K.

Supplementary References

31. Inoue, A. Stabilization of metallic supercooled liquid and bulk amorphous alloys. *Acta Mater.* **48,** 279–306 (2000).

32. Jenčič, I., Bench, M. W., Robertson, I. M. & Kirk, M. A. Electron-beam-induced crystallization of isolated amorphous regions in Si, Ge, GaP, and GaAs. *J. Appl. Phys.* **78,** 974 (1995).

33. Newbury, D. E. & Myklebust, R. L. Simulation of electron-excited X-ray spectra with NIST-NIH desktop spectrum analyzer (DTSA). *Surf. Interface Anal.* **37,** 1045; DOI:10.1002/sia.2086 (2005).
